# Implementation and impact of a point of care electroencephalography platform in a community hospital: a cohort study

**DOI:** 10.3389/fdgth.2023.1035442

**Published:** 2023-08-07

**Authors:** Jared Ward, Adam Green, Robert Cole, Samson Zarbiv, Stanley Dumond, Jessica Clough, Fred Rincon

**Affiliations:** ^1^ Department of Medicine, Division of Critical Care Medicine, Cooper University Hospital, Cooper University Medical School of Rowan University, Camden, NJ, United States; ^2^ Department of Medicine, Critical Care Medicine Fellowship, Inspira Medical Center, Vineland, NJ, United States; ^3^ Cardiopulmonary Department, Inspira Health, Vineland, NJ, United States; ^4^ Department of Neurology, Cooper University Hospital, Cooper University Medical School of Rowan University, Camden, NJ, United States

**Keywords:** status epilepticus, point-of-care EEG, transfers, seizures, community hospital, finances

## Abstract

**Objective:**

To determine the clinical and financial feasibility of implementing a poc-EEG system in a community hospital.

**Design:**

Data from a prospective cohort displaying abnormal mentation concerning for NCSE or rhythmic movements due to potential underlying seizure necessitating EEG was collected and compared to a control group containing patient data from 2020.

**Setting:**

A teaching community hospital with limited EEG support.

**Patients:**

The study group consisted of patients requiring emergent EEG during hours when conventional EEG was unavailable. Control group is made up of patients who were emergently transferred for EEG during the historical period.

**Interventions:**

Application and interpretation of Ceribell®, a poc-EEG system.

**Measurement and main results:**

88 patients were eligible with indications for poc-EEG including hyperkinetic movements post-cardiac arrest (19%), abnormal mentation after possible seizure (46%), and unresponsive patients with concern for NCSE (35%). 21% had seizure burden on poc-EEG and 4.5% had seizure activity on follow-up EEG. A mean of 1.1 patients per month required transfer to a tertiary care center for continuous EEG. For the control period, a total of 22 patients or a mean of 2 patients per month were transferred for emergent EEG. Annually, we observed a decrease in the number of transferred patients in the post-implementation period by 10.8 (95% CI: −2.17–23.64, *p* = 0.1). Financial analysis of the control found the hospital system incurred a loss of $3,463.11 per patient transferred for an annual loss of $83,114.64. In the study group, this would compute to an annual loss of $45,713.05 for an overall decrease in amount lost of $37,401.59. We compared amount lost per patient between historical controls and study patients. Implementation of poc-EEG resulted in an overall decrease in annual amount lost of $37,401.59 by avoidance of transfer fees. We calculated the amount gained per patient in the study group to be $13,936.44. To cover the cost of the poc-EEG system, 8.59 patients would need to avoid transfer annually.

**Conclusion:**

A poc-EEG system can be safely implemented in a community hospital leading to an absolute decrease in transfers to tertiary hospital. This decrease in patient transfers can cover the cost of implementing the poc-EEG system. The additional benefits from transfer avoidance include clinical benefits such as rapid appropriate treatment of seizures and avoidance of unnecessary treatment as well as negating transfer risk and keeping the patient at their local hospital.

## Introduction

A significant proportion of comatose patients in the emergency department (ED) or the intensive care unit (ICU) are at risk of developing nonconvulsive status epilepticus (NCSE), which is defined as a state of continuous or repetitive seizures without convulsions for more than 5 min (1). The annual incidence of status epilepticus (SE) is estimated to be 9.9–41 per 100,000 hospital admissions, with roughly one third of those classified as NCSE (2). Of all patients undergoing EEG in the ICU, 19% have been found to have seizure activity (3) and 48% of patients after convulsive status have been shown to have NCSE (4). Without timely diagnosis, treatment, and extenuation of NCSE, patients are at increased risk of neurological injury and death (5).

Continuous electroencephalogram (cEEG) remains the method of choice to diagnose NCSE with current guidelines recommending initiation within one hour of status epilepticus (1). Unfortunately, EEG is not available at all centers despite its association with improved outcomes due to resources required for its implementation, maintenance, and use (6). This leads to unnecessary transfer to tertiary centers for patients without NCSE resulting in a delay in further evaluation and treatment as well as additional costs and risks to the patient. Even at centers with cEEG, there is frequently a delay in initiation that falls outside the current guidelines (7, 8) An easy to deploy EEG system that allows for rapid diagnosis would fill these voids.

A poc-EEG platform uses fewer EEG leads than traditional EEG but in theory can be applied rapidly and with minimal training. Poc-EEG integrates three main tools for EEG interpretation: (a) raw EEG data, (b) sonification of EEG patterns, and (c) artificial intelligence (AI), which provides a percentage that reflects probability of seizure, obviating the necessity for bedside neurology interpretation. When compared to traditional EEG, poc-EEG has demonstrated similar accuracy in diagnosing NCSE (9, 10). Poc-EEG has been successfully implemented in academic centers allowing for timely and accurate assessment of patients in the critical care setting (8). A case series of 10 patients has shown the successful use of poc-EEG to assist in timely diagnosis and treatment of suspected seizure in a community hospital (11).

**Figure 1:**
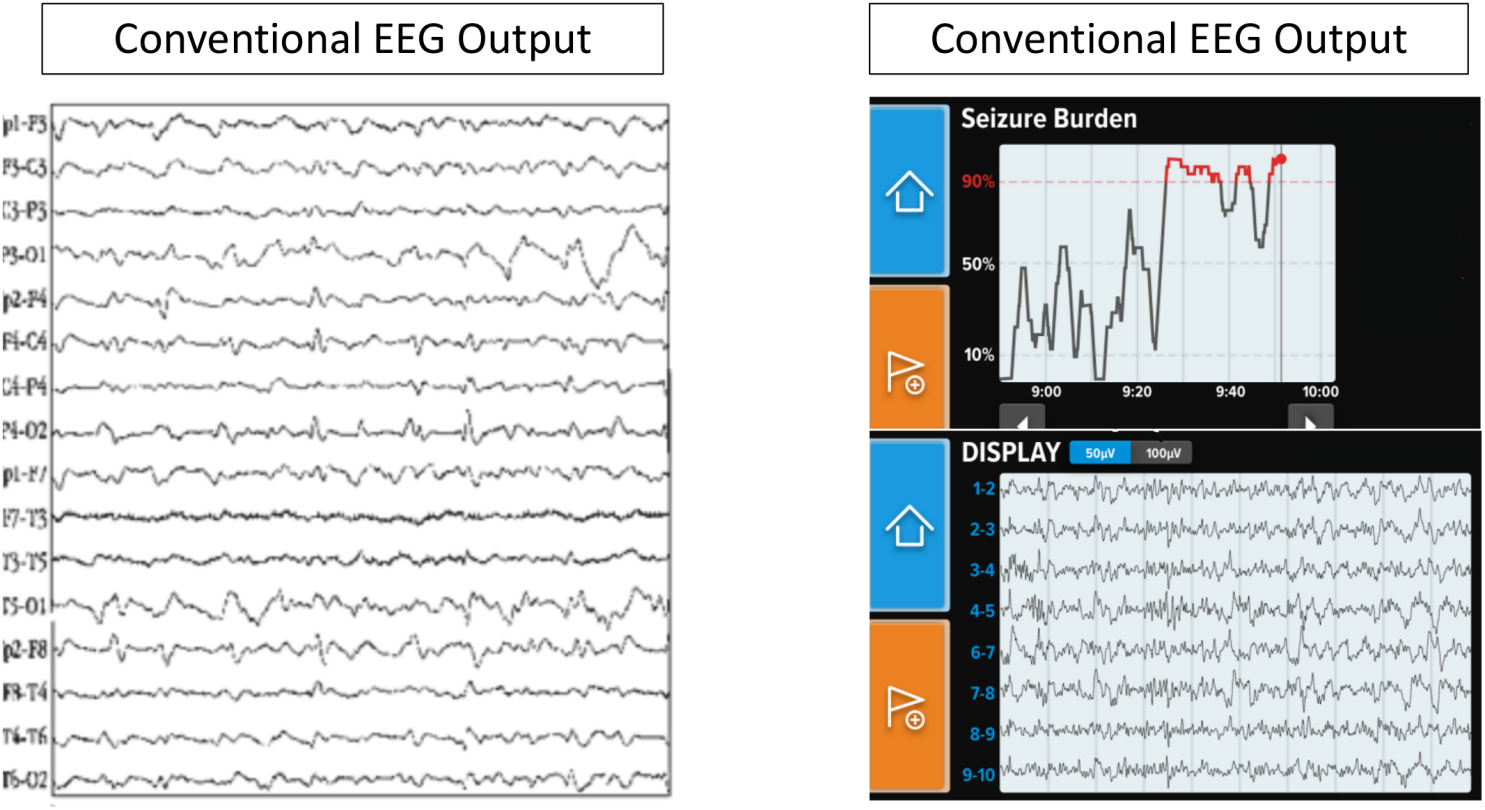
Conventional EEG vs Ceribell EEG.

One poc-EGG system, Ceribell® (Mountain View, CA), is an FDA-approved limited montage 10 electrode EEG system that can be rapidly applied to patients with a suspicion of seizure. To ensure appropriate connectivity, each electrode has a gel which is expelled and after twisting the external part of the electrode a green light is displayed. Once all ten electrodes have an adequate connection, the device bedside monitor begins recording. Ceribell's® software algorithm used by the Seizure Detection module identifies sections of EEG that may correspond to electrographic seizures via preprocessing and segmenting signals into smaller events and then evaluating those signals based on time, frequency and channel features over a moving 5-min window. If the seizure thresholds are reached the device produces an alarm. The algorithm generated a seizure alert with 100% sensitivity if burden >50%, 88% if >10% but more importantly showed a negative predictive value of 99% if no seizure burden was reported (12). A comparison of Ceribell and conventional EEG characteristics is provided in Table 1 and Figure 1.

**Table 1:** Ceribell vs. Conventional EEG characteristics.

	Conventional	Ceribell
Frequency		
Sampling rate (range)	200–1,000 Hz	250 Hz
Frequency response (range)	0.01–500 Hz	0.5–100 Hz
Channels	32	8
Number of electrodes	21	10

In this study, we investigated the feasibility of implementing Ceribell®, a poc-EEG system, in a community hospital. We analyzed the effect of poc-EEG on clinical and financial outcomes. Specifically, our objectives were to: (a) describe implementation of a poc-EEG system at a community hospital; (b) characterize a study cohort who would undergo poc-EEG; and (c) provide basic analysis of potential cost benefit from transfer avoidance and apply that analysis to the cost of the technology.

## Methods

### Study design

Patients were prospectively identified with concern for seizure activity necessitating EEG for management during hours when conventional EEG was not available. This included patients with abnormal mentation potentially due to NCSE or rhythmic movements with concern for underlying seizure activity. A retrospective cohort consisting of patients requiring transfer for emergent EEG were used to determine the baseline number of patients transferred monthly and the costs associated with the transfer.

### Setting

Two community hospitals within the Inspira Health Network located in Vineland, NJ (262 beds) and Mullica Hill, NJ (210 beds) were identified. Neurology consultation was available at both hospitals as well as routine spot EEGs during regular business hours, specifically 9am–5pm with limited neurology coverage and no EEG technicians on weekend days. Twenty-four-hour critical care fellow support was available at both hospitals as well as intensivist daytime coverage and 24-hour availability. Data was collected between January and October 2021. Historical data was obtained from the calendar year 2020. Transfers during the month of December 2020 were excluded as this is when the poc-EEG device was piloted, which affected the number of transfers. The year preceding intervention was chosen as the historical cohort for several reasons. While practice patterns throughout the COVID-19 pandemic likely changed, the two years more closely resembled each other than if compared to a period not within the COVID-19 pandemic. Furthermore, prior to 2020 neurology consultation within the hospital system was even more sporadic as it relied on outpatient neurology coverage. Starting in 2020, a dedicated in-patient neurology consultant was hired to cover normal business hours. Prior to this year, patients were likely transferred for neurology consultation alone, making comparison impossible.

### Intervention

**Figure 2:**
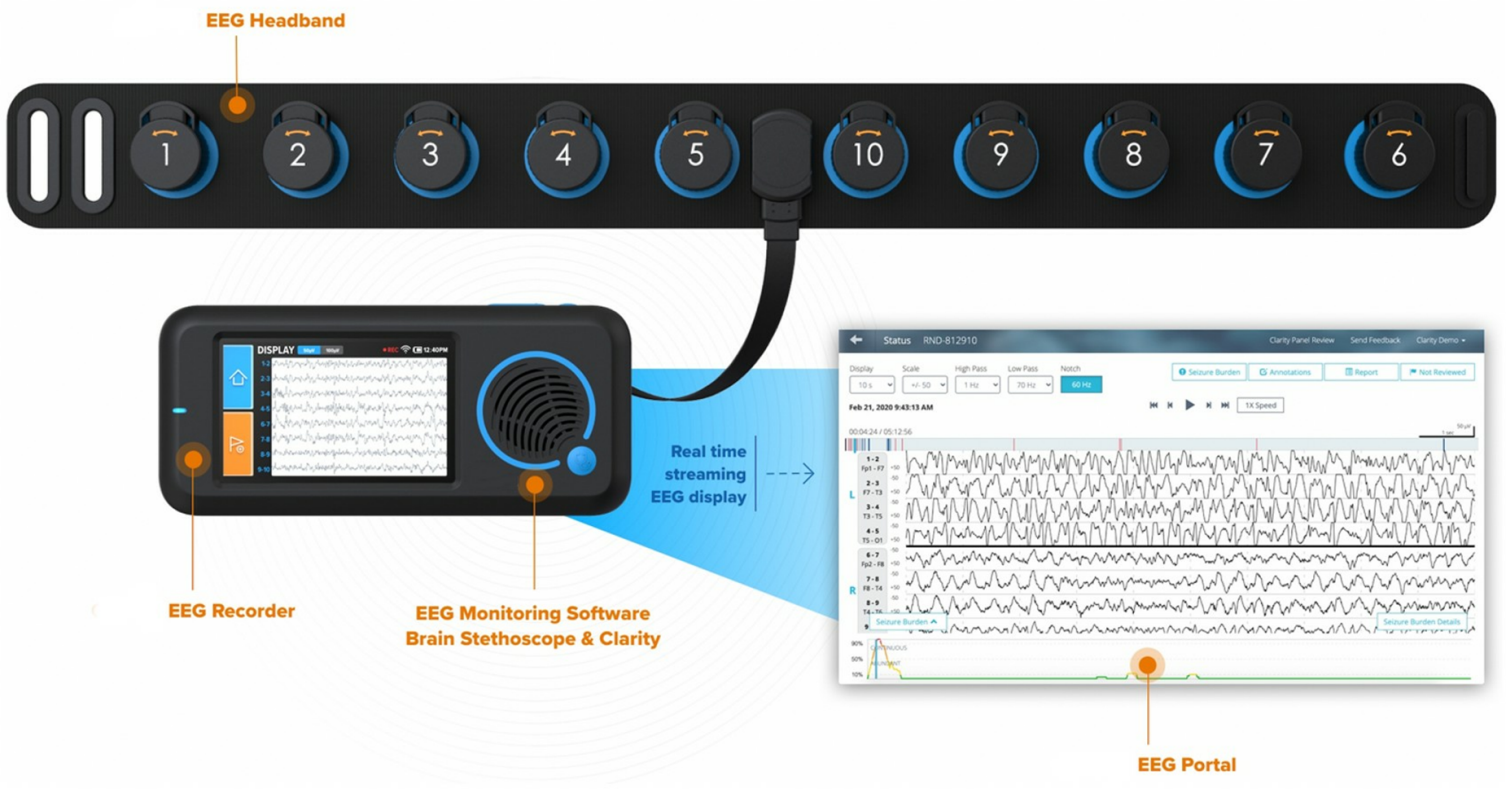
Ceribell poc-EEG headband, recorder and portal.

Ten critical care fellows were trained in how to appropriately apply the Ceribell® headband (Figure 2) by the company's educator. Each fellow had a unique login for the mobile portal, allowing for further investigation. No training was needed by the bedside nurse, other than to notify the fellow if the device alarmed or connectivity failed (red light displayed).

**Figure 3:**
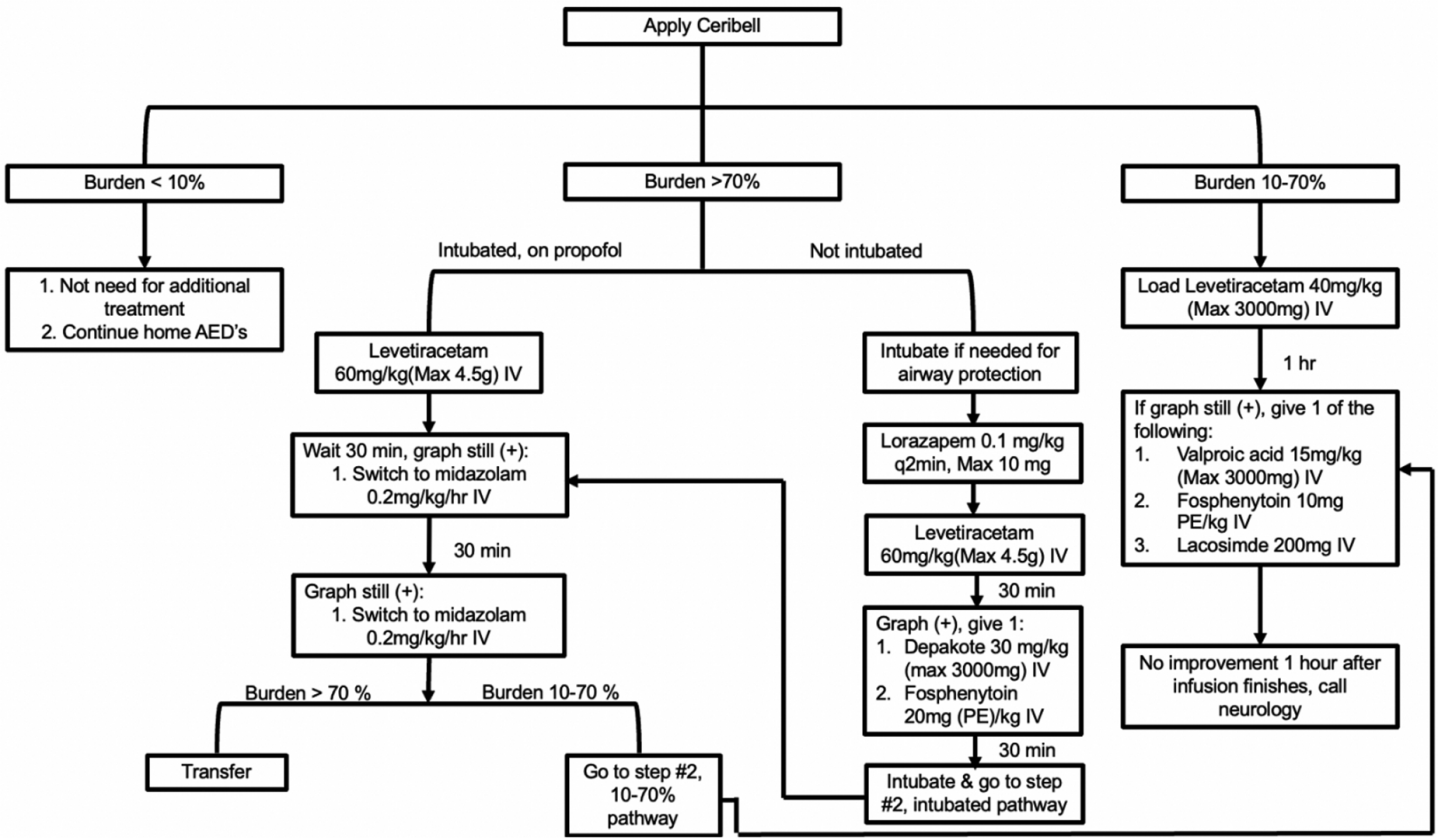
Treatment algorithm.

**Figure 4:**
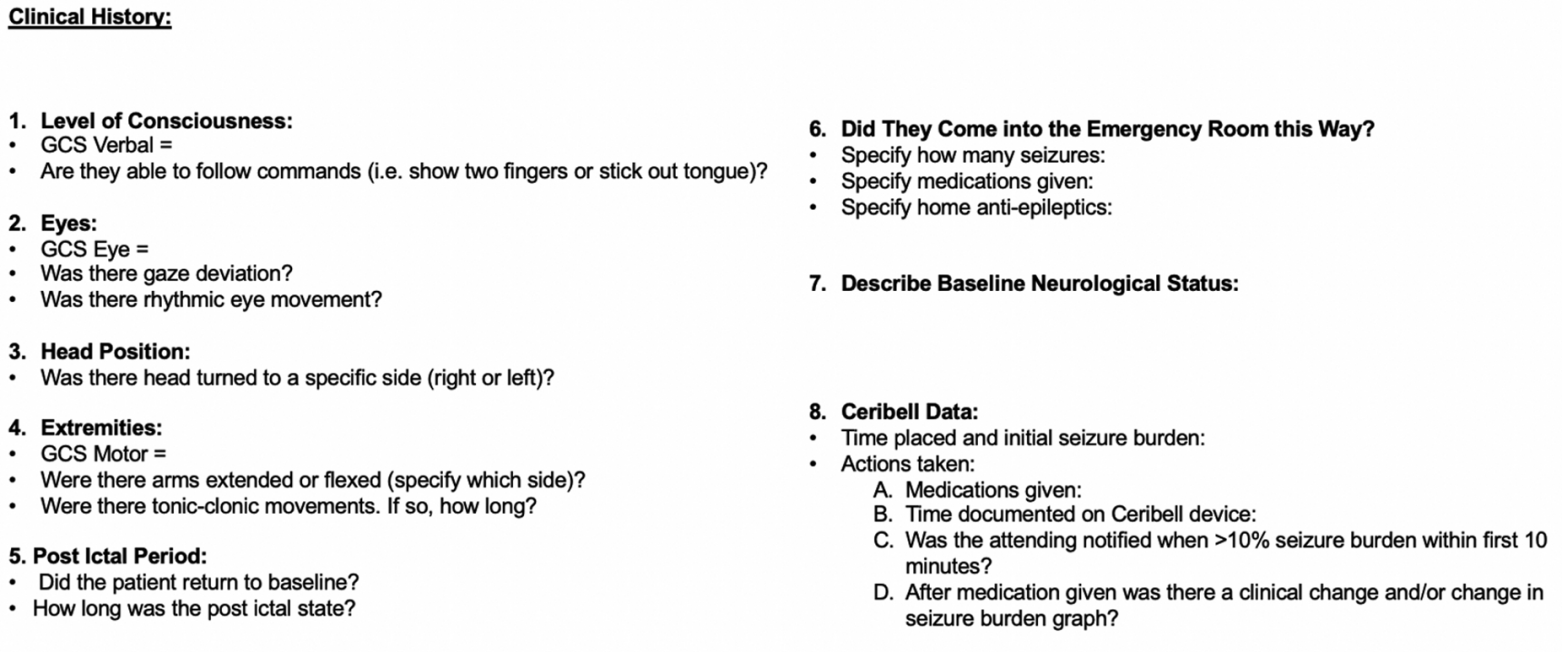
Poc-EEG event note.

**Figure 5:**
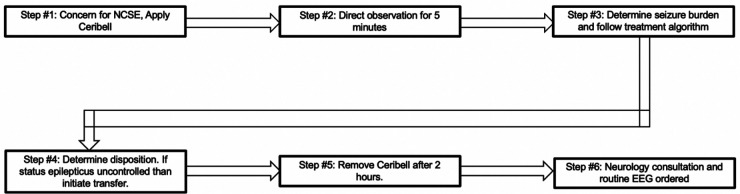
Poc-EEG workflow.

Patients in the ED or ICU with concerns for seizure activity were prospectively identified and Ceribell® headband device was applied. These patients demonstrated either abnormal mentation possibly due to NCSE or rhythmic movements potentially due to convulsive seizure activity. After application, the critical care fellow directly observed the patient for the first five minutes. The EEG headband was then allowed to record for a maximum of two hours. After removal of the headband, the critical care fellow would review the seizure threshold reached during the entire two hours via the online portal. A treatment algorithm was provided (Figure 3) that instructed the appropriate treatment intervention if the seizure threshold was <10%, 10%–70%, or >70%. Intensivist and neurology consultants were available if needed. Based on the findings and intervention required, a standardized note was documented (Figure 4). During the next regular business hours, a standard EEG was performed on all patients as well as a neurology consultation. In addition, the poc-EEG tracings were interpreted by the neurology consultant. Figure 5 describes the workflow.

### Population

**Figure 6:**
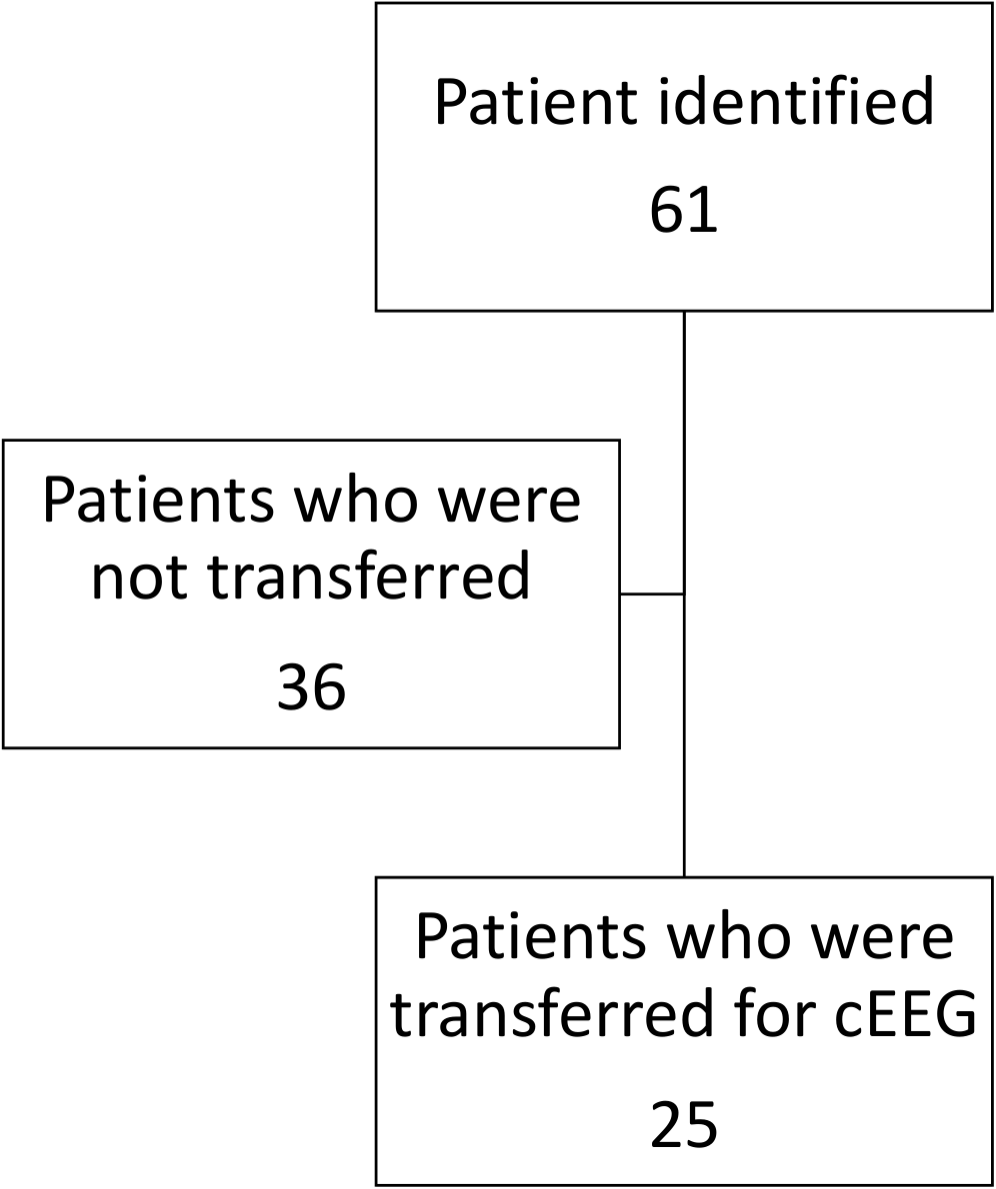
Poc-EEG cohort.

After adopting the use of poc-EEG, clinical criteria were established pertaining to appropriate use. Its use was restricted to patients requiring emergent diagnostic EEG during hours when conventional EEG was not available. The use of poc-EEG was left to the treating physician's preference and was not part of a study protocol. Common indications included patients with hyperkinetic movements post-cardiac arrest, patients with history of seizures and/or witnessed convulsive seizure activity without return to baseline mentation, and all other patients found unresponsive or stuporous upon admission with concern for NCSE (Figure 6). The study size was determined by the total number of patients receiving poc-EEG.

The control group consisted of patient transferred for emergent EEG during the historical period.

### Data collection

Prior to data collection, the institutional review board of Inspira Medical Center approved the study (2022-02-001) and waived the need for informed consent. The standardized note was reviewed as well as the neurologists' dictation of the poc-EEG study and the corresponding standard EEG. Their official interpretation was used to determine electrographic seizure activity as well as response to treatment. Chart review was then completed to identify disposition outcome, mortality, and complications that occurred throughout the hospitalization.

After the patient was discharged and/or transferred, a coding summary was generated by the financial billings department and was included in the patient's medical record. Each patient's hospitalization record was reviewed, and the billing department provided the average net billed and accrued.

### Financial analysis

Understanding the financial feasibility after implementation of a new product is important for its longevity. The focus of the analysis was to define the financial impact due to the absolute reduction in the number of transferred patients after implementation of poc-EEG. We chose to compare transfers to outside hospitals for emergent EEG between groups. While this may not capture the total financial impact of poc-EEG implementation, it can act as a surrogate reflecting the financial burden on the health care system. Transfers to outside hospitals have been linked to increasing health care costs, and, at least for NCSE, these costs have been shown to be decreased through the use of technology and AI (13, 14). Further analysis could be done to determine if the cost of the technology could be covered due to patient transfer avoidance.

For all analysis, we used the mean amount collected for each group. We did not have access to financial data at the level of individual patients. We used the amount collected as opposed to the amount charged as charged amounts are subject to pricing differences across institutions and therefore may limit generalizability of results.

To determine the financial impact, we did the following. First, we calculated the annual loss due to patients transferred for EEG. We took the control group and determined the mean collected per patient. We subtracted the expenses of transfer from this value. This represented the net loss per patient transferred. This could then be extrapolated to an annual loss based on total number of patients transferred during the control year. We assumed that there were no differential transfer costs between transferred patients in both groups. This allowed us to take the same amount lost per patient and apply it to the number of patients transferred in the treatment group providing an annual loss after implementation of poc-EEG. The difference between these two values represents the decrease in annual loss of patients requiring transfer for emergent EEG.

In order to determine the number of patients who avoided transfer needed to cover the fixed cost of the device, we did the following. We took the mean amount collected for the treatment group and subtracted the variable cost of the headband. We then subtracted the amount lost if the patient was transferred (calculated above). This represented the net earned by avoiding patient transfer. We then calculated the annual fixed cost of the Ceribell® system (monthly subscription fee × 12). By dividing, the annual cost of the technology by the amount earned per patient avoiding transfer, the number of transfers needed to cover the expense of the system could be determined.

### Statistical analysis

The results are mostly descriptive in this study. Comparison of frequencies was preformed using the Chi Square tests. 95% confidence interval was used with statistical significance at *p* < 0.05. Quantitative data including reduction in transfers and financial outcomes did not require further analysis. Confidence intervals for financial analysis were unable to be calculated as only the mean for each group was collected. All data was accounted for.

## Results

### Clinical characteristics

**Figure 7:**
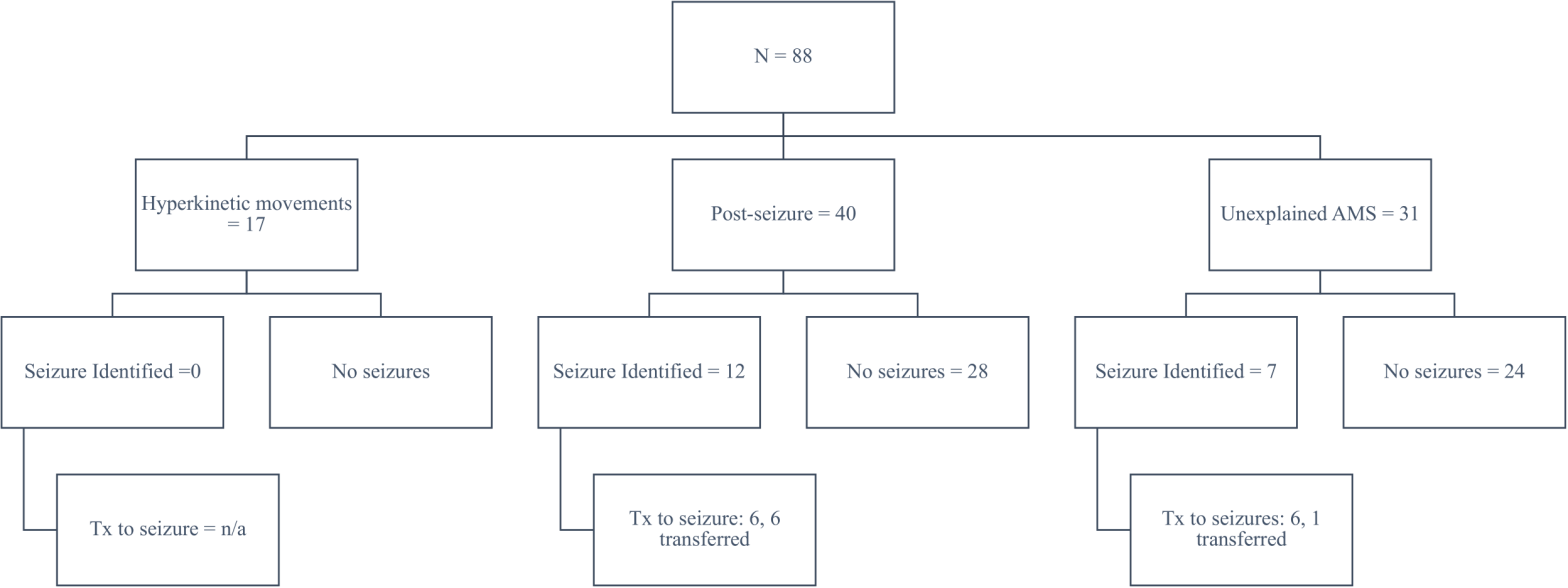
Poc-EEG cohort characteristics.

From January through October 2021, we implemented and used poc-EEG in 88 subjects. Eligible subjects included patients with hyperkinetic movements post-cardiac arrest (19%, *n* = 17/88), patients with a history of seizures and/or with witnessed convulsive seizure activity and without return to baseline (46%, *n* = 40/88), and all other patients found unresponsive or stuporous upon admission to the hospital with concern for NCSE (35%, *n* = 31/88). Approximately 10% (*n* = 9/88) of the poc-EEG were applied in the emergency room, the rest of the patients were identified in the ICU (90%, *n* = 79/88). Of the 88 patients, 21% (*n* = 19/88) had significant electrographic seizure burden on poc-EEG and 4% (*n* = 4/88) had electrographic seizure activity confirmed on follow-up EEG. Another 5% (*n* = 5/88) were transferred immediately after poc-EEG identified high burden of electrographic seizure activity as an immediate need for continuous EEG was identified; therefore poc-EEG excluded and/or decreased ongoing concern for electrographic seizure activity in 78% (*n* = 69/88) of our inception cohort (Figure 7).

Demographic characteristics of the patients in this cohort showed a mean age of 57 years old (95% CI: 53.27–60.65), 52% (*n* = 46/88) were male, 46.5% (*n* = 41/88) female, and 1 person identified as transgender (gender not identified). Approximately 16% (*n* = 14/88) had a history of seizures on AEDs. Overall, the cohort was 64% Caucasian (*n* = 56/88) with 20% African American (*n* = 18/88), 11% Hispanic (*n* = 10/88), 0.3% Asian (*n* = 3/88), and 0.1% other (*n* = 1/88). All data was accounted for without any missing variables. (Table 2).

**Table 2:** Demographic characteristics.

	Cohort characteristics	Historical characteristics
Age (years)*	57 (53.27, 60.65)	58 (50.08, 67.84)
Gender		
Male	52.2%	60.9%
Female	46.6%	39.1%
Transgender	1.2%	0%
Race		
Caucasian	63.6%	56.5%
African American	20.5%	17.4%
Hispanic	11.3%	26.1%
Asian	3.3%	0%
Other	1.2%	0%

* Data presented as mean (25th percentile, 75th percentile).

Only 2 patients where poc-EEG identified 0% electrographic seizure burden were found to have electrographic seizure activity on the follow-up standard EEG; thus only 2.4% of patients (*n* = 2/83) were found to have electrographic seizure activity on follow-up EEG despite a negative poc-EEG; 5 patients were transferred before follow-up EEG could be performed.

### Transfer data

During the study period, eleven patients (mean of 1.1 per month) were transferred for emergent EEG. This constituted 13.4% (*n* = 11/82) of the total cohort. During 2020, 22 patients (mean of 2 per month) were transferred to a tertiary center for emergent EEG. The difference between these two values represents the decrease in the annualized number of patients requiring transfer for emergent EGG. This computed to an annual estimate of 10.8 patients (95% CI: −2.17–23.64, *p* = 0.1).

### Financial analysis

The control group had a mean amount collected of $4,036.89 per patient. This is for the initial treatment and stabilization of the patient prior to transfer. For our hospital system, most patients requiring transfer go to one academic hospital that is roughly 40 miles away. Given the acuity of illness of these patients and need for ACLS trained nursing and appropriate monitoring capabilities, the transfer center reported a mean cost of $7,500 per patient billed to the sending facility. When adjusted for the amount collected per patient, this result in a mean loss of $3,463.11 per patient or an estimated annual loss of $83,114.64. This was calculated by multiplying $3,463.11 by 24, the number of patients transferred during the control period.

When the study period is analyzed, the eleven patients who required transfer would result in a loss of $38,094.21, $3,463.11 per patient, for the ten-month study period or estimated annual loss of $45,713.05. For this cohort after the implementation of poc-EEG, Inspira experienced an overall decrease in amount lost due to transferred patients of $37,401.59. This was calculated by subtracting $37,401.59 from $83,114,64.

The treatment cohort (those that received poc-EEG) had a mean collection of $11,161.33 per patient. As above, assuming each patient transferred incurs a loss of $3,463.11 per patient every patient kept because of poc-EEG would result in a net positive of $13,936.44 per patient after the cost of the poc-EEG headband was applied. The poc-EEG system has a monthly fixed cost of $9,975 for a multi-hospital system or $119,700 annual cost. To cover those costs, 8.59 patients per year (0.72 per month) would need to avoid transfer. We demonstrated a reduction in transfer of 0.9 patients per month (Table 3). It would take 9.5 months to recover upfront costs. The number needed to avoid transport to recuperate annual costs would be significantly lower if that patient required flight transport as these costs often exceed $40,000.00 and therefore would make avoidance of unnecessary transfer more important.

**Table 3:** Poc-EEG net income.

	Revenue (per patient)		Variable expenses (per patient)	Net income (per patient)
	Billed	Collected		
Control	$11,361.30	$4,036.89	$7,500.00 (transfer cost)	Control Collected–Transfer cost = −$3,463.11
Ceribell	$28,585.49	$11,161.33	$688.00 (headband cost)	Ceribell collected–headband =$10,473.33
Savings by avoiding transfer		Ceribell net income – Control net income = $13,936.44		
Ceribell annual fixed cost		Ceribell monthly cost ($9,975.00) × 12 months = $119,700.00		
# of prevented transfers needed to cover costs		($119,700.00)/($13,936.44) = 8.59		

## Discussion

Critically-ill neurological patients account for at least 10%–15% of admissions to intensive care units of which 8%–34% will experience seizure activity (15, 16). Approximately 3.3% of all critically ill patients experience seizures and a high index of suspicion needs to be had by providers especially in comatose patient or those without return to baseline mentation (17). Of seizures captured in one study, 34% were nonconvulsive seizures, and of these, 76% were NCSE (3). Emergent EEG has been noted to be of increasing importance in critical care but access to this diagnostic modality has remained limited. At one large US tertiary care medical center, where EEG availability and accessibility barriers should be minimal, the time to EEG in the ICU was 3.5 h (7). However, outside of these centers, even that time is unachievable as one study showed that in 286 emergent EEGs, the average interval from request to formal reporting was 1.13 days (18). A recent publication of the use of poc-EEG in COVID-19 patients showed that for 10 consecutive device applications, mean time to interpretation was 23.8 min compared to 126.5 min for routine 18-channel studies (19). Before the advent of poc-EEG, many smaller hospitals would often transfer patients for these services; one study conducted in 24 West Virginia hospitals found that the need for critical care and neurology services accounted for nearly 54% of all transfers during their study period (20). Thus, there is clearly a need for and adaptation of poc-EEGs aimed at reducing the overall time to EEG as well as expanding EEG availability outside of tertiary care centers but data on this is limited. Poc-EEG also has the added benefit of faster application and exposure to those applying the device to patients who may have communicable diseases.

Our experience provides a pragmatic framework on how to successfully implement this technology in a community setting with limited neurological coverage. The logistics regarding proper implementation and use of poc-EEG is often the largest obstacle to overcome. The stepwise approach provided here may provide guidance for other institutions with similar EEG availability and a means to fill that void. In addition, the data provided here demonstrates this can be done with a high concordance between poc-EEG and the following standard full montage EEG. This leads to improvement in care provided and a decrease in the absolute number of transfers to tertiary centers.

Avoidance of unnecessary transfers allows patients to be cared for in their own community. This decreases the burden of travel on the patients' family. It allows provides an opportunity for the patients' outpatient providers to continue to participate in the patients' care. This also eliminates risk associated with transfer. While the analysis did not show a statistically significant reduction in transfers, it did show an absolute reduction in number of transfers and a favorable financial analysis. The study was under powered and results could vary if examined on a larger scale. The analysis included provides financial justification for implementation of poc-EEG systems. Previous studies showed that transfers to referral centers are associated with higher costs to patients and often with no changes in treatment management (14). Transferred patients hospital cost were on average $9,600 higher compared with non-transfer patients and a recent US study showed transfer costs of $6,160 plus a $24.64 per mile charge for ground transport and $11,760 for air transfers, which excluded billing for other services (14, 21).

The major benefits of adapting poc-EEG include improved clinical care by addressing a diagnostic deficit (i.e., access to EEG during off hours). Other benefits, such as promoting patient satisfaction and minimizing transfer risk, are also notable. Financial analysis supported the cost of the implementation. This was done by examining per patient average collection as well as calculating the amount saved by minimizing transfer costs. While these savings did not cover the entire cost of the technology there is additional financial benefit from avoiding transfer. While this is challenging to calculate retrospectively as it is difficult to identify which patients would have been transferred if poc-EEG was not available. We were able to calculate on average how much each poc-EEG patient collected and from that determine how many patients needed to avoid transfer to cover the costs of the poc-EEG. Given the ease of use and absolute reduction in transfers between the two cohorts, poc-EEG will likely justify its associated cost and reduce out of hospital transfers.

Poc-EEG demonstrated a very low false negative rate for patients with minimal electrographic seizure activity on poc-EEG but confirmed electrographic seizure activity on standard EEG. Explanation for the false negatives could be attributed to the time between studies. False positives or those with electrographic seizure activity on poc-EEG but in fact negative standard EEG would be hard to identify in our study design. As all patients with positive poc-EEG would warrant anti-epileptic treatment and thus an explanation for the follow up negative standard EEG. There would need to be concurrent poc-EEG and standard EEG. An understanding of the outcomes of those transferred would add strength to our data.

Additionally, poc-EEG provide reliable data. As previously stated, a study showed 88% sensitivity for seizure burden >10% and 100% sensitivity if >50% but more importantly, a 99% negative predictive value if seizure activity was not identified by the device (12). Furthermore, when compared to conventional EEG, Ceribell® showed equivalent signal quality and durability (10). This previously published data mirrors our own experience of a low false negative rate (2.4% in our cohort) and leads to our determination that the poc-EEG device can be safely implemented in the community hospital setting.

## Limitations

The retrospective nature of the historical cohort makes the data less granular than desired. Outcome measures, EEG findings, and reason for transfer can be hard to determine from chart review. We are confident that the main indication for transfer for both groups was need for emergent EEG, however there is always room for potential error. This limits comparison between the groups.

Given the retrospective design, we were limited in our ability to capture all drivers of transfer. An understanding of the treatment plan at the tertiary hospital and the outcomes of the EEGs at those hospitals is lacking from our data. The use of the poc-EEG was left to the treating physician's preference which introduces selection bias. While we believe the historical and study group have very similar patient demographics and severity of illness, it is possible there are seasonal or other changes that occur that are not recognized. A more robust data collection plan would have allowed for adjustment of both chronic conditions such as history of seizure or neurological injury as well as aspects of the acute illness that may have influenced the decision to transfer. This would have allowed for a more confident comparison between the groups. Both groups occurred during the COVID-19 pandemic, and this may have unknown effects. As the pandemic progressed, practice patterns changed which could influence decisions on transfer thus effecting our results.

The finances associated with treatment costs vary based on location, insurance, and many other factors. If more hospitals from diverse settings participated, the generalizability of the financial analysis would have increased. The cost cited in this study may not be replicated exactly by other institutions. In addition, more formal financial analysis could be implemented on future similar studies.

## Conclusion

Our study highlights the continued importance for community medical centers to develop ways to provide rapid diagnosis and treatment for patients at risk of status epilepticus. This study is the largest study that shows the use of poc-EEG in a community setting and how it can lead to a decrease in unnecessary transfers with potential reduction in hospital costs.

## Prior presentation

A portion of the work will be presented at SCCM Conference 2022; however, this manuscript has not been published elsewhere and is not under consideration by another journal.

## Summary statement

Point-of-care EEG can be implemented in a community hospital with a high degree of diagnostic accuracy preventing transfers to tertiary centers with a very favorable financial profile.

## Data Availability

The raw data supporting the conclusions of this article will be made available by the authors, without undue reservation.
